# Serum Levels of TNF Receptor Ligands Are Dysregulated in Sepsis and Predict Mortality in Critically Ill Patients

**DOI:** 10.1371/journal.pone.0153765

**Published:** 2016-04-28

**Authors:** Christoph Roderburg, Fabian Benz, Florian Schüller, Ines Pombeiro, Hans-Joerg Hippe, Norbert Frey, Christian Trautwein, Tom Luedde, Alexander Koch, Frank Tacke, Mark Luedde

**Affiliations:** 1 Department of Medicine III, University Hospital RWTH Aachen, Pauwelsstrasse 30, 52074, Aachen, Germany; 2 Department of Internal Medicine III, University of Kiel, Schittenhelmstrasse 12, 24105, Kiel, Germany; University of Louisville School of Medicine, UNITED STATES

## Abstract

**Introduction:**

TNF superfamily members, including TNF-related weak inducer of apoptosis (TWEAK) and Glucocorticoid-Induced TNFR-Related Protein Ligand (GITRL) have been described as serum based biomarkers for inflammatory and immune mediated diseases. However, up to now the role of TWEAK and GITRL has not been analyzed in critical illness and sepsis.

**Methods:**

GITRL and TWEAK serum concentrations were measured in 121 critically ill patients (84 fulfilled with septic disease), in comparison to 50 healthy controls. Results were correlated with clinical data.

**Results:**

Serum levels of TWEAK and GITRL were strongly decreased in critically ill patients compared with healthy controls. Concentrations of TWEAK (but not GITRL) were further decreased in patients with sepsis and correlated with routinely used markers of inflammation and bacterial infection such as C-reactive protein, procalcitonin and Interleukin-6. Notably, we failed to detect a correlation to other TNFR ligands such as TNF or APRIL. Finally, TWEAK levels of the upper quartile of the cohort were prognostic for mortality during ICU treatment.

**Conclusion:**

TWEAK and GITRL levels were lower in intensive care unit medical patients. Levels of TWEAK were further decreased in septic patients, and alterations in TWEAK concentrations were linked to an unfavorable outcome. Together with recently published results on other TNFR ligands, these data indicate specific functions of the different TNFR ligands in septic diseases.

## Introduction

Sepsis and septic shock syndrome are characterized by a highly regulated release of a broad variety of cytokines and chemokines. The tumour necrosis factor (TNF) alpha was among the first soluble proteins factors described in the context of septic disease. Recently other members of the TNF superfamily were shown to fulfil a similar role than TNF in the pathophysiology of infectious or inflammatory diseases [1]. A proliferation-Inducing Ligand (APRIL) has been implicated in the pathogenesis of critical illness and sepsis [2]. APRIL serum concentrations are elevated in critically ill patients [3], and elevated APRIL levels were suggested to be diagnostic for septic disease and were even demonstrated to be predictive for patients survival in the context of critical illness [4]. Moreover, APRIL concentrations were correlated to tumour necrosis factor concentrations in critically ill patients and predicted patients’ outcome with even higher accuracy than TNF. However, the pathophysiological implications of these findings and especially the functional role of TNF superfamily ligands in critical illness and sepsis are not clearly understood.

Similar to APRIL, the Tumor Necrosis Factor Weak Inducer of Apoptosis (TWEAK) is a member of the TNF/TNFR superfamily. TWEAK was first described to induce apoptosis in a human adenocarcinoma cell line [5]. Displaying a much wider tissue distribution than TNF, it has previously gained attention as a pivotal regulator of inflammation and cell death in many cells and pathological conditions [6–9]. TWEAK exists as a membrane-bound form and as a soluble variant, formed after proteolytical cleavage by endoproteinases. Both TWEAK-variants exert their biological activity after binding to the Fibroblast growth factor-inducible 14, representing their bona fide receptor. Alterations of TWEAK serum concentrations have recently be described in the context of various inflammatory and cardiovascular diseases [10–12].

Another TNF/TNFR superfamily member, Glucocorticoid-Induced TNFR-Related Protein Ligand (GITRL), was identified simultaneously with its receptor in 1997 [13]. In humans, GITRL expression was described in a broad panel of immune cells such as antigen-presenting cells, monocytes and B cells as well as in endothelial cells [14, 15]. In response to various inflammatory stimuli, these cell-types were shown to rapidly up-regulate GITRL mRNA. In line, several studies provided evidence for a role of the GITR/GITRL system in regulating inflammation. As such, a pro-inflammatory role of GITRL and its receptor was demonstrated in a mouse model of acute lung and colon injury. Moreover, the GITR/GITRL system was shown to play a regulatory role in leukocyte extravasation [13–16].

Despite the emerging roles of TWEAK and GITRL in inflammation and immunity, no data on a function of these ligands are involved in severe systemic infection in humans are available. To analyse the regulation of TWEAK and GITRL in critically ill patients and to evaluate their potential diagnostic and prognostic potential, TWEAK and GITRL serum levels were measured in 121 critically ill patients at the time point of admission to a intensive care unit.

## Materials and Methods

### Study design and patient characteristics

TWEAK and GITRL serum levels were analyzed in a recently published cohort of 121 critically ill patients (71 male, 50 female; median age 66 years, range 18 to 90 years; Table 1) (nur das Osteopontin). Patients’ information and samples were acquired prospectively. Follow-up was performed as recently described [17]. Diagnosis of sepsis, severe sepsis and septic shock was performed according to the criteria proposed by the ACCP/SCCM Consensus Conference Committee. Patients that did not fulfill these criteria were categorized as non-sepsis patients [18, 19]. As a control population, we analyzed 50 healthy blood donors (35 male, 15 female, median age 37 years, and range between 18 and 67) free of chronic diseases like diabetes, cardiac and pulmonary diseases and with normal values for blood counts, C-reactive protein and liver enzymes. Patients´ blood was taken ad admission to the ICU and in some cases after 3 days of treatment. Samples were handled on ice, sera were frozen at -80°C. Routine laboratory and experimental parameters were measured as described previously [18, 20–22].

**Table 1:** Baseline patient characteristics.

Parameter	all patients	non-sepsis	sepsis
Number	121	37	84
Sex (male/female)	71 / 50	23 / 14	48 / 36
Age median (range) [years]	66 (18–90)	59 (18–84)	68 (20–90)
APACHE-II score median(range)	19 (3–40)	17 (4–25)	19 (3–40)
SAPS2 score median(range)	43 (9–80)	42 (13–80)	43.5 (9–65)
ICU days median (range)	9 (1–137)	7 (1–41)	12 (1–137)
Death during ICU n(%)	24.0%	21.6%	25.0%
Death during ICU orfollow-up n(%)	47.0%	45.7%	47.5%
Mechanical ventilationn(%)	88 (72.7%)	24 (64.9%)	64 (76.2%)
Ventilation time median(range) [h]	120.5 (0.0–2966.0)	49,5 (0.0–986.0)	146,7 (0.0–2966.0)
pre-existing diabetes n(%)	32.8%	28.6%	34.6%
WBC median (range) [x10³/μl]	12.7 (0.1–208)	12.0 (1.8–29.6)	13.0 (0.1–208)
CRP median (range)[mg/dl]	112 (<5–230)	17 (5–230)	181 (<5–230)
Procalcitonin median(range) [μg/l]	1.0 (0.0–125.2)	0.1 (0.1–36.5)	1.9 (0–125.2)
Interleukin-6 median(range) [pg/ml]	75 (0–6100)	29 (7.6–260)	94 (0–6100)

APACHE, Acute Physiology and Chronic Health Evaluation; CRP, C-reactive protein; ICU, intensive care unit; SAPS, simplified acute physiology score; *WBC*, *white blood cell count*

The study protocol was approved by the local ethics committee and conducted in accordance with the ethical standards laid down in the Declaration of Helsinki (ethics committee of the University Hospital Aachen, RWTH-University, Aachen, Germany, reference number EK 150/06). Written informed consent was obtained from the patient, his or her spouse or the appointed legal guardian.

### Determination of TWEAK and GITRL serum concentrations by ELISA

We determined TWEAK and GITRL serum concentrations by using a commercially available enzyme immunoassay (ELISA, both: Ray Biotech, Norcross GA, USA).

### Statistical analysis

Statistical analysis have been performed as recently described in detail [18, 20–22]. In brief, data are given as median and range to reflect the skewed distribution of analysis on human samples. The Mann-Whitney- U-test and for multiple comparisons the Kruskal-Wallis-ANOVA was used. Box plot graphics display a statistical summary of the median, quartiles, range and extreme values. Correlations analysis were performed by using the Spearman correlation tests. The prognostic value of the variables was tested by univariate and multivariate analysis in the Cox regression model. Kaplan Meier curves were plotted to display the impact on survival. ROC curves were generated by plotting sensitivity against 1-specificity. All statistical analyses were performed with SPSS (SPSS, Chicago, IL, USA) [23, 24].

## Results

### TWEAK and GITRL serum concentrations in critically ill patients

**Fig 1:**
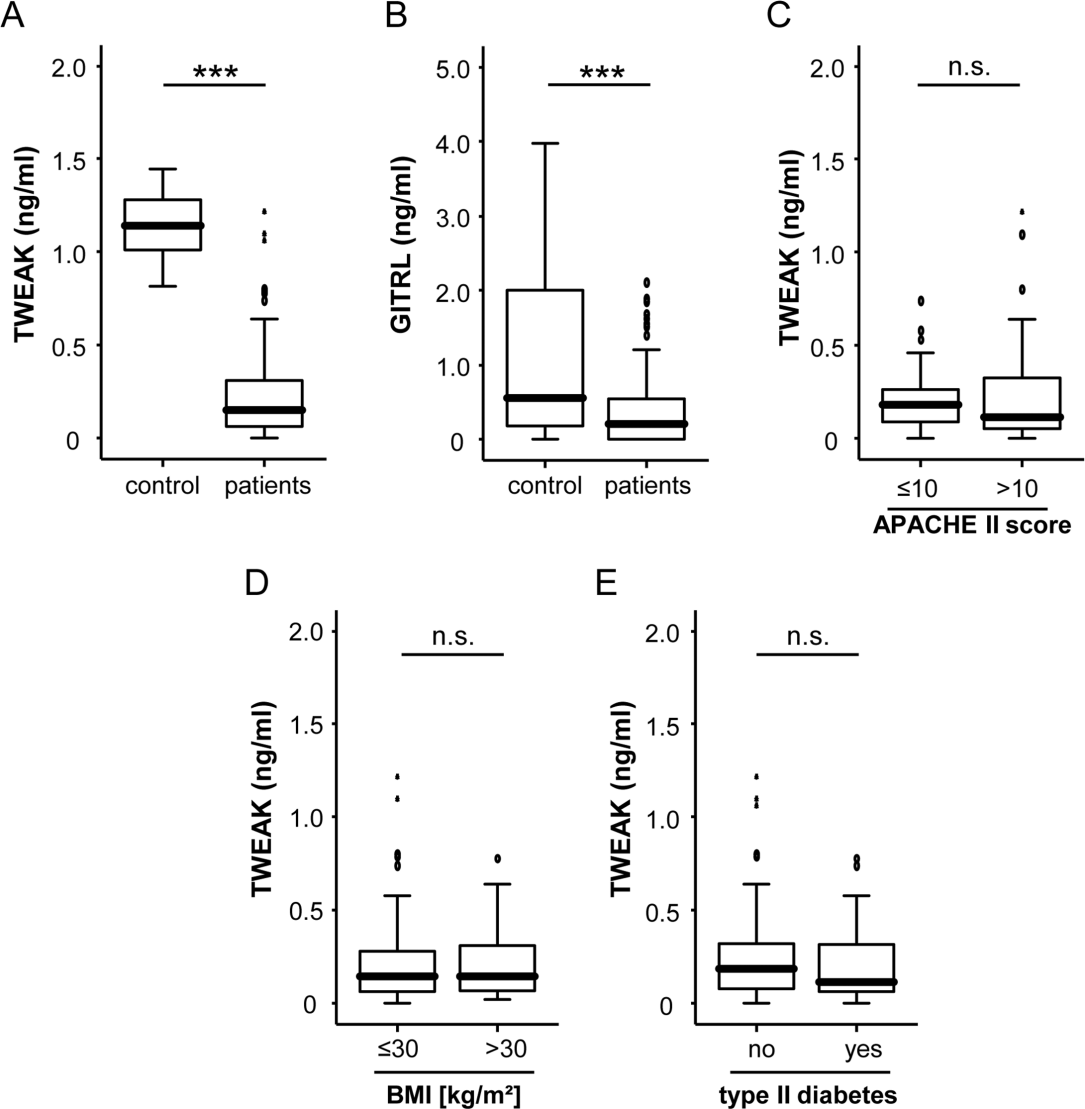
Serum TWEAK and GITRL concentrations of critically ill patients at ICU admission. (a,b) TWEAK and GITRL serum levels at admission to the ICU were significantly (P < 0.001, U-test) lower in critically ill patients (n = 121) compared to healthy controls (n = 50). (c) TWEAK concentrations at admission to the ICU were independent on the severity of disease. (d) Serum TWEAK concentrations were similar in critically ill patients with or without obesity. (e) Serum TWEAK concentrations were similar in critically ill patients with or without diabetes mellitus typ 2. *** p< 0.001

To analyze the significance of these circulating TNF superfamily ligands in critical disease, we measured concentrations of TWEAK and GITRL in sera of 121 critically patients as well as in 50 healthy volunteers by using commercially available ELISA. In contrast to previously published data on TNF and APRIL [2], serum levels of TWEAK were significantly lower in the patients´ group, when compared to the control group (Fig 1A). Similar to TWEAK, the median level of GITRL was lower in critically ill patients than in healthy controls (Fig 1B). However, serum concentrations of GITRL were overall very low and partly close to the detection limit of the assay and we therefore did not pursue it in further analysis as as it could not been excluded that any subsequent regulations may be only artefactual.

Notably, TWEAK levels were independent of the stage of disease (Fig 1C). Considering the well-known association between TNF superfamily ligands and the metabolic status of patients [25], we next analyzed potential correlations between TWEAK and body mass index (BMI) or the presence of type 2 diabetes. However, TWEAK serum levels were independent on the presence of obesity (Fig 1D) or type 2 diabetes (Fig 1E). Finally, TWEAK serum levels did not differ when patients were analyzed with respect to their age or gender (Table 1 and data not shown).

### TWEAK serum levels are decreased in patients with sepsis

**Fig 2:**
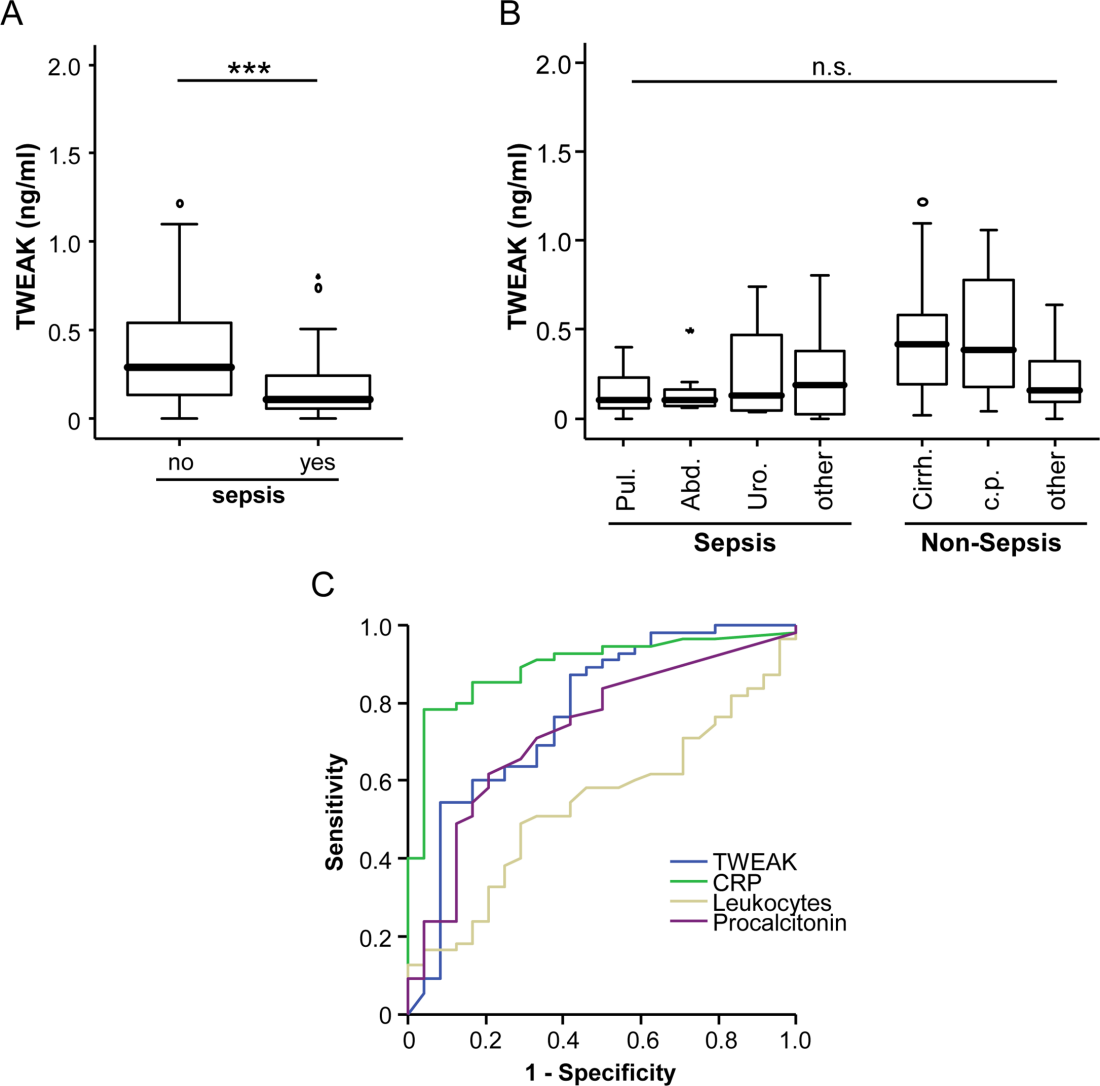
Serum TWEAK concentrations are decreased in sepsis. (a) Critically ill patients with sepsis (n = 84) displayed significantly lower TWEAK serum concentrations (n = 37, U-test) compared to patients without sepsis. (b) TWEAK serum concentrations were not different in patients with different etiologies of critical illness and are highest in patients with cardiac diseases. (c) ROC curve analyses comparing the diagnostic power in predicting sepsis of TWEAK with well-established laboratory markers: C-reactive protein (CRP), procalcitonin, and white blood cell count (leukocytes).

We next analyzed the influence of bacterial infection and sepsis on TWEAK serum levels. TWEAK serum concentrations were decreased in ICU-patients that fulfilled the criteria for septic disease(Fig 2A). We next performed subgroup analysis to understand whether a deregulation of TWEAK might be specific for certain disease etiologies. Among the 84 patients that fulfilled sepsis criteria in our study, 53 were categorized as pulmonary sepsis, 11 abdominal sepsis, 4 urogenital sepsis and 16 as septic disease with a different or unknown infectious focus. Among the non-septic patients, 13 suffered from cardiopulmonary diseases, 10 suffered from decompensated liver cirrhosis and 14 had another diagnosis leading to critical illness (Table 2). Notably, by comparing TWEAK (Fig 2B) concentrations between these distinct patients´ groups we did not find a specific regulation, corroborating that decreased TWEAK is a general feature of sepsis.

**Table 2:** Disease etiology of the study population.

	sepsis	non-sepsis
	n = 84	n = 37
**Etiology of sepsis critical illness** Site of infection n (%)		
Pulmonary	53 (63.1%)	
Abdominal	11 (13.1%)	
Urogenital	4 (4.8%)	
Other	16 (19.0%)	
**Etiology of non-sepsis critical illness** n (%)		
cardiopulmonary disease		13 (35.1%)
decompensated liver cirrhosis		10 (27.0%)
non-sepsis other		14 (37.8%)

To further confirm the link between decreased TWEAK concentrations and septic disease we performed correlation analysis revealing that TWEAK concentrations closely correlated to markers of infection in these patients (CRP, PCT and IL-6; Table 3). Comparative ROC-curve analysis on the predictive power for sepsis of TWEAK concentrations and other routine laboratory parameters revealed that TWEAK displayed superior or equal area AUC-statistics compared to leucocyte or PCT, but inferior AUC values compared to CRP (Fig 2C).

**Table 3:** Correlations of TWEAK serum concentrations at admission day with other laboratory markers.

	ICU patients	
	TWEAK	
Parameter	r	p
***Markers of liver function***		
Protein	0.229	0.026
Albumin	0.221	0.054
INR	0.035	0.711
AST	0.264	0.006
Bilirubin	0.075	0.505
GLDH	0.227	0.024
***Markers of inflammation***		
CRP	-0.276	0.002
Procalcitonin	-0.205	0.048
IL-6	-0.359	0.021
TNF-alpha	-0.056	0.732
***Markers of renal function***		
Creatinine	0.045	0.632
Urea	-0.059	0.528
***Others variables***		
Lactate	0.210	0.028
Resistin	-0.259	0.054
LDH	0.169	0.066
Ventilation time	-0.212	0.062
Overall survival days	-0.375	0.006
ICU days	-0.261	0.004
***Clinical scoring***		
APACHE-II	-0.073	0.495

r, correlation coefficient; p, p-value; r and p-values by Spearman rank correlation

### TWEAK concentrations are associated with ICU survival

**Fig 3:**
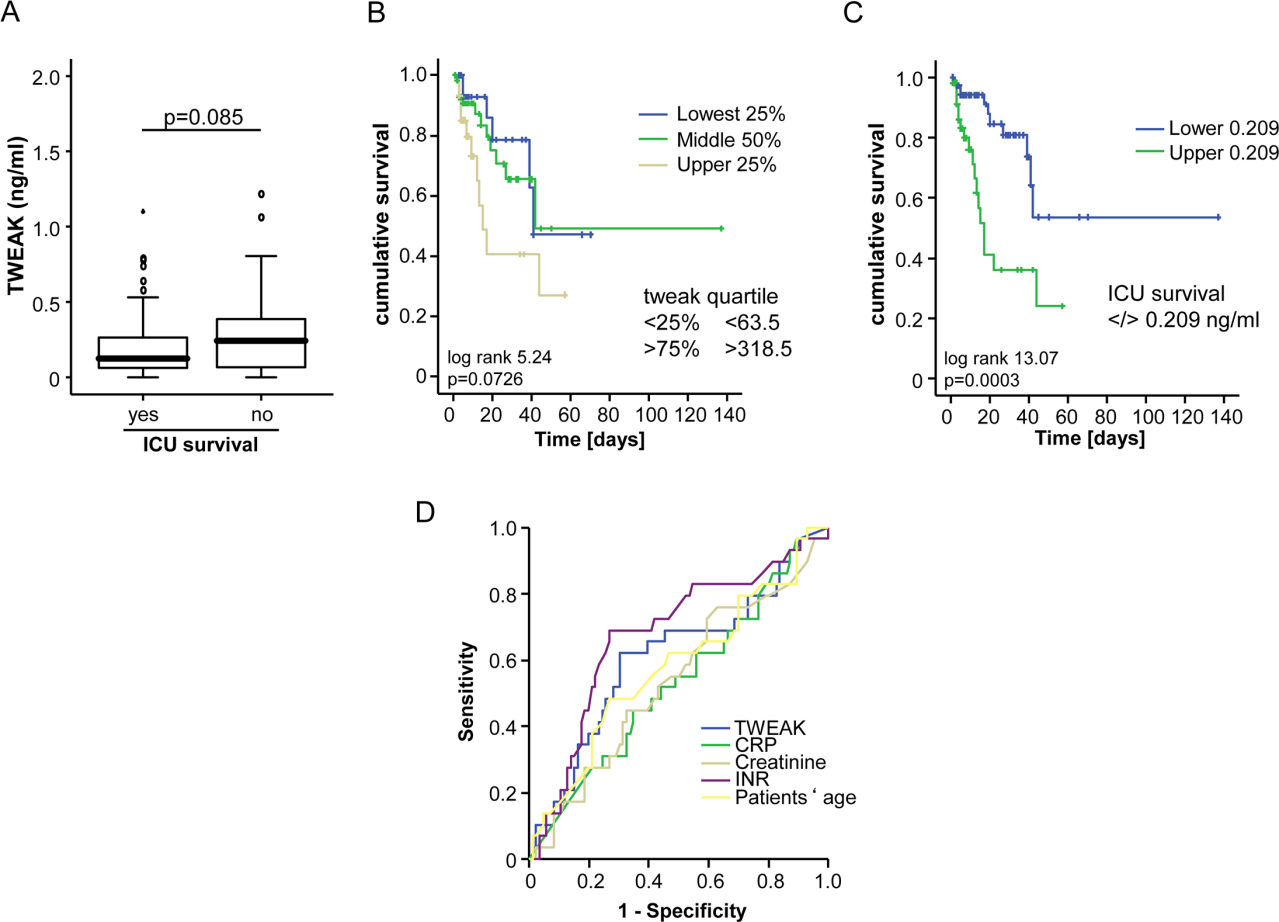
Prediction of ICU mortality by TWEAK serum concentrations. (a) Patients that succumbed to death during ICU treatment displayed higher TWEAK serum levels compared to survivors. (b) Kaplan-Meier survival curves of ICU patients showed that patients with low TWEAK levels an increased short-term survival at the ICU. (c) Kaplan-Meier survival curves of ICU patients revealed that patients with TWEAK levels < 209 ng/ml had an increased ICU survival. (d) ROC curve analyses comparing the prognostic value of TWEAK at admission for ICU survival with the patients´ age and routinely used laboratory parameters.

To identify a potential association between TWEAK serum concentrations and patients´ outcome during and after ICU treatment we first compared the concentrations in patients that succumbed to death during ICU treatment and those that survived ICU treatment (Table 1). Notably, serum concentrations of TWEAK were significantly different between these subgroups of patients (Fig 3A). Patients who survived ICU treatment displayed significantly higher TWEAK levels than those patients that succumbed to death (Fig 3A). We next analyzed the prognostic accuracy of TWEAK serum concentrations and therefore performed Cox regression- and Kaplan Meyer curve analyses. These analyses confirmed the unexpected observation that those patients that displayed high TWEAK levels (e.g. TWEAK levels within the upper quartile of the ICU cohort) had a significantly worse prognosis than the others (Fig 3B). Further analyses revealed that the prognostic value of TWEAK was slightly inferior to INR but superior to CRP, creatinine and patients`age according to ROC curve analysis. Moreover, we applied the Youden-index method to define the best cut-off value to discriminate survivors from ICU non-survivors. This analysis revealed that patients with TWEAK levels of lower than 209 pg/ml had a significantly better prognosis compared to the other patients (Fig 3C). We finally applied ROC curve analyses to compare the prognostic value of TWEAK measurements at admission for ICU survival with that of routinely used laboratory parameter (Fig 3D).

**Fig 4:**
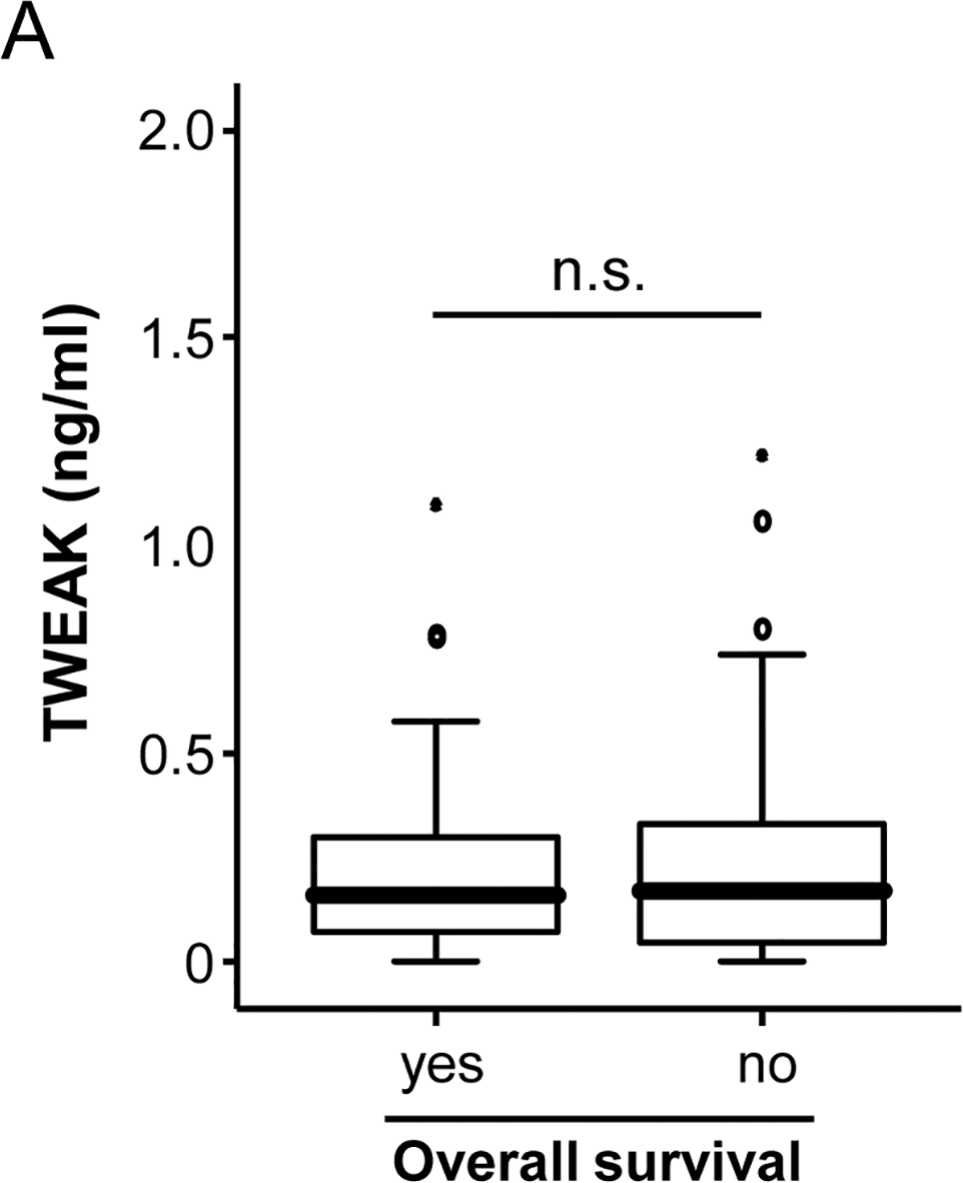
Prediction of long-term survival by analysis of TWEAK serum levels. (a) TWEAK levels were unaltered in critically ill patients that survived in the long term follow up compared to patients that succumbed to death (U-test).

During the follow-up period, 24% of the patients died on the ICU and additional 23% of the patients died after discharge from the ICU. We therefore tested whether TWEAK serum concentrations at admission to ICU could also predict long-term survival. However, patients that died during long-term follow up had a tendency towards higher TWEAK levels than survivors (Fig 4A). Of note, in this setting statistical significance was failed (p = 0.062), which may suggest that patient´s long-term survival is determined by a complex interaction of a network of different factors rather than a single parameter, which is in line to the results of a previous analysis on this patient group [2].

**Fig 5:**
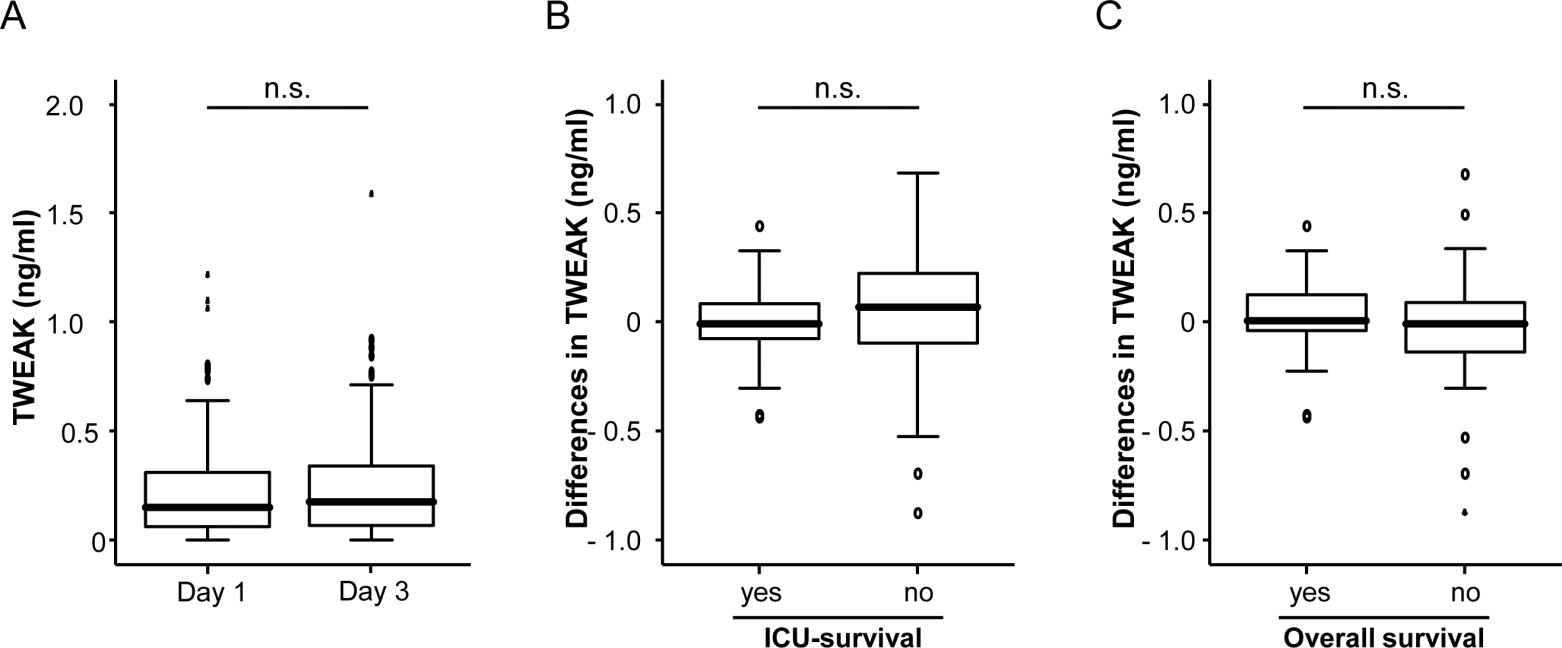
Time course of TWEAK concentrations in critically ill patients. (a) TWEAK serum concentrations are unchanged in critically ill patients at admission to the ICU and after three days of ICU treatment (U-test). (b, c) TWEAK serum concentrations were determined at admission to the ICU and after three days of ICU treatment. The differences in TWEAK levels between these time-points were not predictive for ICU or long-term survival.

Considering the link between ICU survival and TWEAK serum levels, we next examined whether kinetics of TWEAK serum concentrations during the first three days of ICU treatment might indicate patients prognosis. Importantly, longitudinal analysis of TWEAK concentrations revealed no relevant change between the time point of admission and day 3 of ICU-treatment (Fig 5A). Consequently, the differences between admission and day 3 did not reflect patients´ prognosis during or after ICU treatment (Fig 5B and 5C).

### Comparison of serum TWEAK, serum APRIL and tumor necrosis factor concentrations

**Fig 6:**
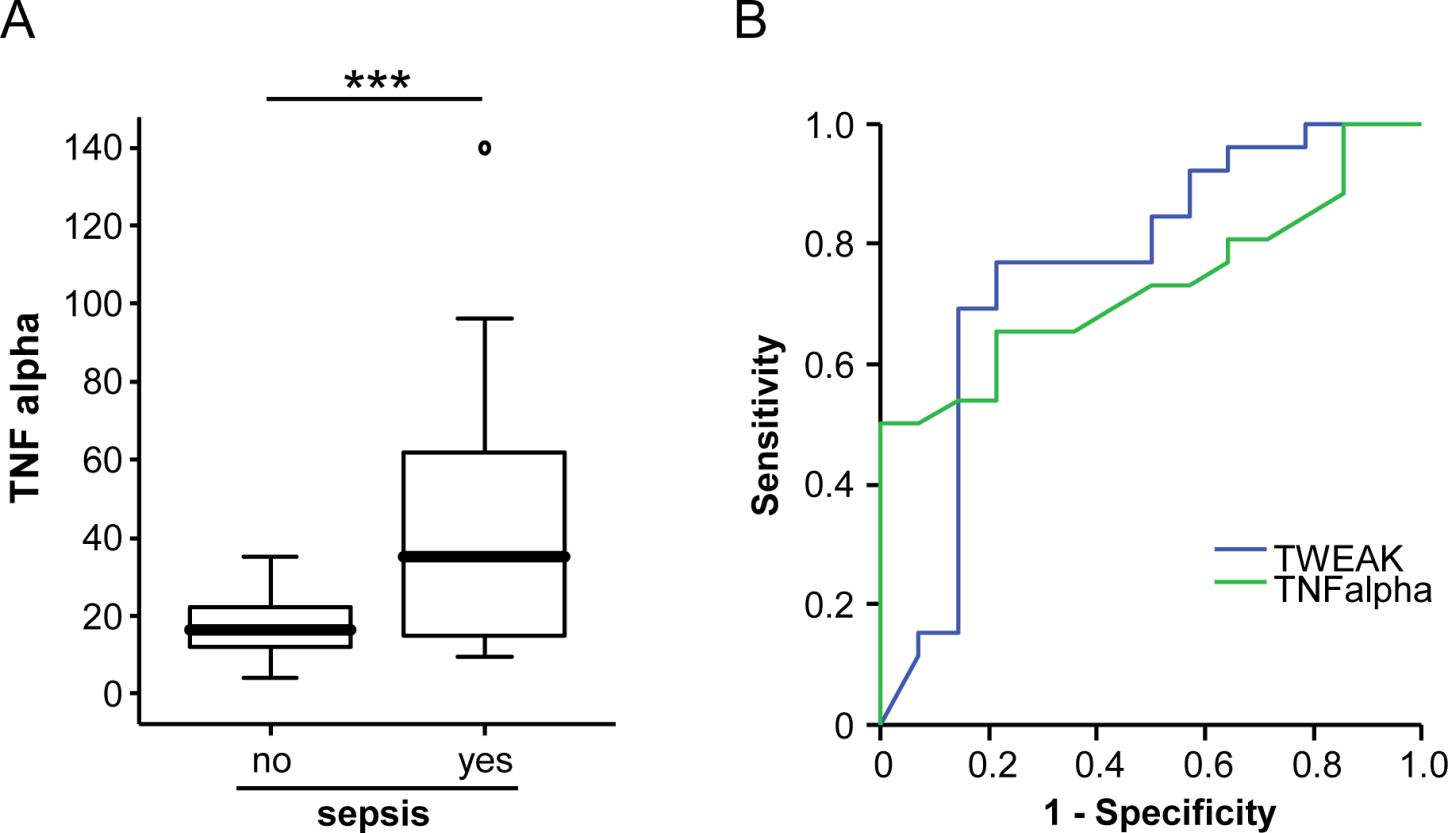
TNF and TWEAK levels for the diagnosis of sepsis. (a) Patients that fulfilled sepsis criteria had higher TNF levels when compared with critically ill patients without sepsis. (b) ROC analyses comparing the diagnostic power of TWEAK and TNF for sepsis.

We recently demonstrated a clear correlation of serum levels of TNF and APRIL in critically ill and sepsis patients [2]. We now attempted to compare these markers with TWEAK and GITRL in the more general context of critical illness. These analyses revealed a clear upregulation of TNF (Fig 6A) in sepsis patients. Based on the clear regulations of TNF and TWEAK in sepsis, we next performed correlation analysis between TNF and TWEAK. However, within the 40 patients with available measurements for both markers, we failed to detect a correlation between TNF and TWEAK (Table 3). When comparing the diagnostic accuracy for sepsis, TWEAK displayed a slightly superior AUC value (0.762) compared to TNF (0.731; Fig 6B). Finally, levels of TWEAK and TNF demonstrated a similar accuracy for prediction of patients’ fate during ICU treatment (not shown).

## Discussion

TNF has been closely associated with the pathogenesis of sepsis [26, 27], and increased levels of TNF were found in the serum of patients with sepsis [3, 4], suggesting that other members of the TNF superfamily might also server as biomarkers in this context. Similar to TNF, we recently demonstrated that elevated APRIL serum levels can be found in patients with sepsis and are associated with a poor prognosis. In the present study we analyzed serum concentrations of circulating TWEAK and GITRL, two other members of the TNF superfamily in critically ill and septic patients. We demonstrate decreased TWEAK serum levels in a large, well characterized cohort of critically ill patients admitted to our ICU. TWEAK levels correlated with markers of inflammation and infection and were further decreased in patients with sepsis. In contrast to our results, Louise Schilder and coworkers recently reported significantly increased TWEAK serum levels in septic patients [28]. We cannot fully explain the apparent discrepancy between the report of Schilder et al. and our study. It may–at least in part- be explained by different group sizes and differences in the experimentsl design (n = 42 in the report of Schilder et al. with n = 18 patients meeting criteria for sepsis vs. n = 121 in our report, of whom 84 fulfilled criteria for sepsis, no healthy control group in the study of Schilder et al.). Moreover, all patients in the report of Schilder et al. received continuous hemofiltration due to acute renal failure, while in our collective of critically ill patients renal failure was only one of several entities that caused critical illness. So maybe further studies on the impact of renal function on serum TWEAK levels are needed to clarify the discrepancies between the work of Schilder et al. and our report.

In contrast to previous results of TNF and APRIL serum levels in this cohort, TWEAK and GITRL serum levels were not elevated. Conversely, TWEAK levels were significantly lower compared with healthy controls. Moreover, we could not demonstrate a clear correlation between TWEAK/GITRL serum levels and disease severity according to the APACHE-II-score. Additionally, while TNF, APRIL and TWEAK serum concentrations reflected the presence or absence of sepsis in critically ill patients we failed to detect a similar correlation for GITRL. These striking differences most likely reflect significant biological differences in their biosynthesis and mechanisms of release into the serum: TNF and TWEAK are both pro-inflammatory cytokines that are able to induce cell death in different tissues. However, while TNF expression and serum release is upregulated during inflammation [29] data from animal experiments suggest that TWEAK is stably expressed in multiple tissues and downregulated on mRNA level in different disease models of sepsis and autoimmune diseases [30]. Although there is an inverse relation between TWEAK mRNA levels and inflammation in these mouse models, the chronological connection between loss of TWEAK and severity of pathological manifestations is not fully understood. In line with these results from mouse models, our patients with critical disease show a decrease in serum TWEAK levels compared to healthy controls. However, in these patients, higher TWEAK levels correlate with a poor prognosis. We cannot fully explain this effect. Our data might reflect the complex role of the inflammatory response during the disease progress of patients suffering from sepsis or SIRS due to other causations (e.g. cardiogenic shock or severe hepatic disease) [2, 31]. Profound inflammatory dysbalance is a pathologic commonality of many critical diseases. One might speculate that while serum TWEAK levels are basically reduced in critically ill patients, different levels of reduction reflect the magnitude of inflammatory imbalance, which might strongly affect the patients`outcome. Thus, our data sound a note of caution that therapies intended to augment TWEAK responses in critically ill patients could carry a significant risk.

Further molecular analyses are clearly needed to more specifically assess the inverse regulation and function of TNF and TWEAK in critical disease. For example, the proinflammatory factor NFκB is a potent executor of both TNF and TWEAK signaling. In heart failure, it has been suggested that differential activation of canonical and non-canonical NFκB signaling might determine different effects of both cytokines [7]. It remains to be assessed if NF-κB also mediates differential roles of TNF and TWEAK in critical ill patients due to other causes.

In contrast to TWEAK, GITRL was not differentially regulated in critical ill patients vs. controls. Off note, our negative findings on GITRL serum levels with regards to disease severity or distinction between septic and non-septic patients probably reflect specific mechanisms of biosynthesis and release into the serum, but do not preclude an important functional role of the GITRL/GITR system in the mediation of sepsis. GITRL expression is strongly increased in response to proinflammatory stimuli, mainly in antigen presenting cells and endothelial cells [32–35]. Our results demonstrate that this putative up-regulation on a cellular level is not reflected in the serum of critically ill and septic patients. However, this up-regulation in endothelium was suggested to control the extravasation process and leucocyte tissue infiltration [16, 36], critical early steps in the pathogenesis of sepsis and shock. In line, functional experiments revealed crucial functions of the GITR/GITRL system in an animal model of acute lung injury [36]. Based on our findings and the previous data, it might therefore be interesting to evaluate expression levels of GITRL and/or GITR on circulating immune cells, e.g. by FACS, rather than levels of free circulating GITRL in order to demonstrate a correlation of critical illness or sepsis with activation of the GITR/GITRL system.

It has been demonstrated in a variety of the disease models that pronounced therapeutic effects are achievable with soluble TWEAK- and Fn14-specific antibodies [37]. Consequently, TWEAK- and Fn14-specific antibodies are currently tested in phase I studies in patients suffering from rheumatoid arthritis, lupus or solid tumors [37]. Based on the strong upregulation of TNF in sepsis, various trials tested the effect of anti-TNF antibodies (such as infliximab) in critically ill patients with severe sepsis. However, in these trials TNF antibodies failed to detect any beneficial effect. Our data on a differential role of the different TGFR ligands in sepsis might therefore be of special clinical relevance, as they suggest that a more differential interventional approach, that affects specific members of the TNF superfamily could potentially be more successful than the use of TNF antibodies.

Guiding early therapeutic decisions represent important challenges in the treatment of critically ill patients [2]. In light of our findings that decreased TWEAK concentrations are indicative for presence of septic disease and prognosis, our results indicate that measurement of circulating TWEAK levels could be considered as a novel element in the diagnostic algorithm of critically ill patients. Of note, although in our patients the prognostic value of TWEAK serum levels for overall survival was comparable to many well established prognostic markers (inferior to INR, but superior to CRP, creatinine and patients`age) we do not intend to imply that well established clinical scores and standard parameters can be replaced by measuring novel biomarkers like TWEAK, but rather could be complemented. Moreover from a basic science view, our findings highlight differential roles of different TNF superfamily members in the pathophysiology of septic disease. Future studies combining prospective clinical trials and experimental animal models are needed to further establish TWEAK measurements in diagnosis of sepsis and to define the exact role of different TNF superfamily members in the pathophysiology of sepsis.

### Limitations of this study

Since our report is not a case control study, the patient and control are not fully matched regarding sex and age. However, since in our analysis serum TWEAK or GITRL levels were not correlated to patients’ sex or age, it seems unlikely that these factors have biased our analysis. As a retrospective analysis, our study protocol has to face more possible biases like interpretation bias and selection bias [38]. We tried to rule out theses biases as strictly as possible, however we do not intend to imply that our data on TWEAK serum levels in critically ill patients are sufficient to establish this test as new standard in risk stratification of critically ill and septic patients. Further studies on TWEAK are necessary to establish a role of this molecule in this patient group.
